# A prediction model for venous thromboembolism in the intensive care unit: flawed methods may lead to inaccurate predictions

**DOI:** 10.1186/s13054-023-04778-y

**Published:** 2023-12-18

**Authors:** Stephen Gerry, Gary S. Collins

**Affiliations:** Centre for Statistics in Medicine, Nuffield Department of Orthopaedics, Rheumatology and Musculoskeletal Sciences, University of Oxford, Botnar Research Centre, Windmill Road, Oxford, OX3 7LD UK

In their recent article, Guan and colleagues describe the development of a prediction model for venous thromboembolism (VTE) using a large intensive care unit (ICU) multicentre data set from the USA [1]. We appreciate their efforts to produce a machine learning model that is not a typical ‘black-box’, but one where the model output is ‘interpretable’.

However, there are a few points on which we have some concerns. The first point is related to how the model estimates a patient’s predicted risk and whether these estimates are well calibrated. It is important that the output of a prediction model is a probability and not simply a classification (e.g. high risk vs low risk), since probabilities are so much more informative [2]. When a probability is presented to the end user, they are able to apply their own decision threshold. The model presented by Guan and colleagues does appear to produce predicted probabilities, as shown in Fig. 4; however, it is not clear how these probabilities are generated. Predicted probabilities are not typically produced by a random forest model, and therefore, a further stage of analysis is normally necessary. Yet, this is not described. Since the model does appear to output probabilities to the user, it is important that the calibration of the model be assessed in the validation cohort. Calibration is a widely recommended performance measure and recommended in the TRIPOD (Transparent Reporting of a multivariable prediction model of Individual Prognosis Or Diagnosis) reporting guideline [3] and refers to the agreement between a model’s predicted risks and the observed risks. There are several ways to examine calibration; perhaps, the most effective is the calibration plot [4]. However, this paper does not include any assessment of model calibration. The calibration of a model can be impacted by the ‘overfitting’ inherent in the model fitting process, and random forest models are particularly prone to this [5] and are therefore more susceptible to miscalibration. Unless these issues are addressed, it is uncertain whether the risks generated by the model will be accurate or generalizable.

A second point on which we have concern is regarding missing data and the methods that were used to account for it during model development and validation. The authors helpfully describe the proportion of missing data for each variable in Supplementary Fig. 1, and in some cases, the proportion is very high, for example, 45.8% for partial thromboplastin time (PTT). The methods section states that multiple imputation was used to impute missing values. However, it is not clear what method was used to estimate the final model based upon these multiple imputed data sets during model development or to estimate performance statistics during validation. Furthermore, other important information on the imputation approach is missing, such as the number of imputations, the imputation model and whether the outcome was included, and the rationale for assuming that imputation was appropriate [6, 7]. These issues are particularly relevant since the variable with the most missing data, PTT (45.8%), is also the variable with the greatest feature importance. Therefore, a poorly specified imputation model may have a considerable effect on how well the model works, and again, this calls into question the generalizability of the model.

Many of these omissions would likely have been addressed prior to publication had the authors used the TRIPOD reporting guideline, which is a tool to improve the reporting standard of clinical prediction (or diagnostic) models [3]. TRIPOD can already easily be used for artificial intelligence or machine learning models; however, there will soon be an updated version that more explicitly addresses factors that are unique to these types of models [3, 8].

## Data Availability

Not applicable.

## Abbreviations

VTE: Venous thromboembolism
ICU: Intensive care unit
TRIPOD: Transparent Reporting of a multivariable prediction model of Individual Prognosis Or Diagnosis
PTT: Partial thromboplastin time
